# Ethics, values, and law for public health in a world in turmoil

**DOI:** 10.1093/pubmed/fdaf146

**Published:** 2025-12-10

**Authors:** Farhang Tahzib

**Affiliations:** Public Health Ethics and Law Network, Global Network for Academic Public Health; Ethics in Public Health Section European Public Health Association, Utrecht, Netherlands

**Keywords:** ethics, public health, social determinants

***“We are at an inflection point in history. The world is experiencing its biggest shared test since the Second World War. Humanity faces a stark and urgent choice: breakdown or breakthrough. The choices we make—or fail to make—today could result in further breakdown and a future of perpetual crises, or a breakthrough to a better, more sustainable, peaceful future for our people and planet.”***

António Guterres, Secretary-General of the United Nations, Our Common Future^1^

Political leaders, thinkers, and policy makers around the world are calling out that we are at an inflection point in history, with significant opportunities and threats to health and wellbeing of humanity and the planet. In these times, there is a need for public health professionals and institutions to be vigilant and consider their identity, mission, purpose, and the exigencies of these times. What are our values and their implications for practice? What sort of society do we need and want to build? What is our mission and mandate to prevent disease, prolong life, and promote health through the organized efforts of society? What is our role, responsibilities, and lines of action to effectively play our part in building healthy communities and the betterment of the world?

Inflection points are significant moments where established trends change direction potentially leading to radical transformations. Such turning points often herald a new era, spring times of breakthroughs, new possibilities and hope, but also often simultaneously dark winter times of unease, despair, and decay. Established patterns, values, and ways of thinking, and doing things no longer seem to apply. Structures, systems, and institutions appear to be lamentably defective, dysfunctional, and no longer fit for purpose. It is a time of turmoil, uncertainty, and turbulence as vested interests try to cling on to the old order and outmoded ways of thinking. It is a time when the old order needs to be rolled up on a new one put in its stead. As Einstein noted with the case for the shift in thinking with respect to the development of technologies such as atomic weapons, we need “a substantially new manner of thinking if mankind is to survive.”^2^

## The best of times and the worst of times

Charles Dickens in the classic opening words in the Tale of Two Cities used the French Revolution to examine consequences of social injustice and the potential for both destructive violence and transformative change. In describing an inflection point of the time Dickens noted that: *“It was the best of times, it was the worst of times, it was the age of wisdom, it was the age of foolishness, it was the epoch of belief, it was the epoch of incredulity, it was the season of light, it was the season of darkness, it was the spring of hope, it was the winter of despair.”*

The rapid developments in science, technology, and communication have resulted in remarkable improvements in public health and the betterment of the world, with dramatic increases in life expectancy, decreases in infant mortality, with the developments in medicine resulting in treatment and care with wonder drugs, means and methods providing hope for the future. Science and technology have provided a surge in the social evolution of the planet and the means to resolve practical problems of humanity. They have highlighted our interconnectedness and interdependence; an evolution in organized efforts of society towards the inevitable “planetization of mankind”.^3^

Yet “social injustice continues to kill on a grand scale”,^4^ with widening, scandalous inequalities, conflict, war, a climate crisis, and a disunited fragmented world in turmoil. Science and technology have provided even deadlier weapons of war and destruction, with social media communications being abused to create misinformation, hatred, and disunity. Some refer to the global “polycrisis” of our times: when crises in multiple deeply interconnected global systems have become causally entangled in ways that are significantly degrading of humanity’s prospects,^5^ creating misery, suffering, despair, and hopelessness, provoking a frantic search for identity, purpose, and belonging.

**Figure 1:**
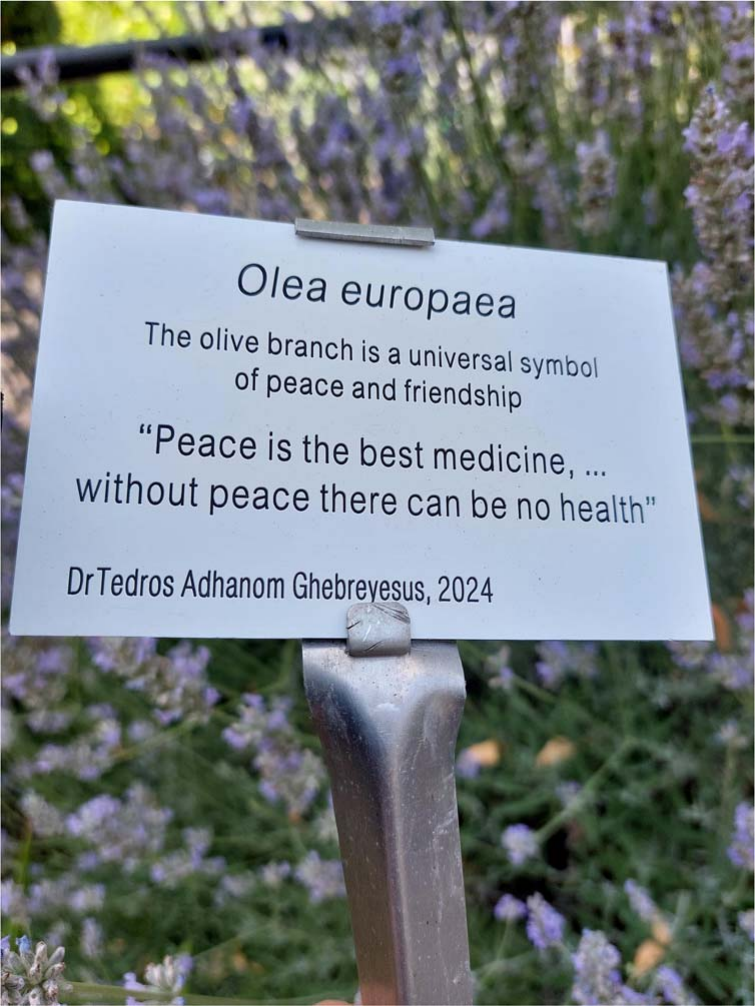
Plaque to mark the International Summit on Ethics, values and Law for good public health policy and practice at Royal College of Physicians, 18 June 2025.

## Decision making for the new age

At this inflection point in history, noted António Guterres, the Secretary-General of the United Nations, “Humanity faces a stark and urgent choice: breakdown or breakthrough.” and that, “the choices we make***—***or fail to make***—***today could result in further breakdown and a future of perpetual crises, or a breakthrough to a better, more sustainable, peaceful future for our people and planet.”^1^

The decisions and choices we make, or fail to make, are not only based on science, statistics, and facts. Our values, beliefs, and the ways we see the world, and want it to be, as individuals, professionals, and institutions, do matter and fundamentally affect our choices, decisions, behaviors, policies, and practice. Public health is driven by its values and is not merely a technical professional discipline concerned with the production of science. Ethics, values, and law matter and are key to good public health policy and practice.

While events, turning points and tipping points are clearly important moments in history, they result from processes and structural transformations over time, which manifest themselves in events. Pandemics, climate change, conflicts and disease are not accidents or misfortunes that merely fall from the sky. They are the results of processes which evolve over decades and then become visible through certain events, or turning points. Therefore it is essential to understand the reality and exigencies of our times and the processes and issues resulting in the poly crisis, the turbulence, and the turmoil. There is a need to understand who we are, where we are, and where we are going so that we can play our role in these times.

## Global solidarity, justice, and moral failures

The science and facts are clear on the nature and impact of climate change, and we have the knowledge, science, and means to ameliorate and address the issue. Yet despite the evidence on the urgency and potential catastrophic consequences, there is a lack of conviction, action, and political will. It is clear that science and technology is not the problem and alone cannot solve the problem.

The COVID-19 pandemic made clear “that pandemic prevention, preparedness, and response reflect choices and value judgements,” and that these decisions are “not a purely scientific or technical exercise, but are fundamentally grounded in ethics.”^6^

The WHO Director-General, in his opening remarks at 148th session of the Executive Board on 18 January 2021, warned of “catastrophic moral failure” if world leaders did not rise to the challenge of vaccine equity^7^ and subsequently referred to it as a test of character, with consequences. A moral failure is an act or thought which is carried out when one knows that it should not be carried out or conversely an act which is not carried out when one knows it should be carried out.

Scholars, in evaluating the responses to the Ebola outbreak and the COVID-19 pandemic, have highlighted that “Nothing will fundamentally change unless we truly understand and appreciate the nature of the lessons we should learn from these outbreaks. Our past failures must be understood as *moral* failures that offer *moral* lessons. Yes, we can learn how to better curb the spread of outbreaks by using shiny new technologies,” it has been suggested, “but unless we appreciate that we have a defect in our collective moral attitude as a global community toward remediating the conditions that precipitate the emergence of outbreaks, we will never truly learn.”^8^^,^^9^

Such moral failures not only result in poor decisions with consequences but also result in moral distress and injury of health professionals.

## Living our stated values

There is a need to improve the ways through which ethical values and commitments are actively incorporated into policy making and day to day public health practice. Ethics, though, has often been considered as an abstract philosophical arm chair exercise, for ethicists to talk among themselves in ivory towers. Sir Michael Marmot, the renowned scholar and advocate on social determinants of health, has noted that he is mystified at why some philosophers “feel no need to engage with non-philosophers” and do not “think that a real life problems are of interest” and seem to be engaged in “highly theoretical discussions that engaged not at all with the real world”.^10^ Some public health professionals and institutions are also often not fully aware of, or even choose to ignore, the inherently ethical nature of many decisions they are considering. They appear to consider ethics as a matter for ethicists or navel gazing, and ethical considerations are often at most an afterthought.

The Public Health workforce, while regularly encountering ethical issues and confronting ethical choices (explicitly and implicitly) in their professional roles, often feel ill-prepared to make “ethical trade-offs,” and must primarily resolve them through personal reflection, with little or no education and training in ethics. They wonder if they have dealt with the ethical issues in the best way, resulting in compounded moral distress.^11^^,^^12^

Also it has been noted that, “unequal power of decision-makers has in many cases undermined the ethical path forward. Because solidarity, equity, transparency, and global justice are ethical values there is a likelihood these values will be ignored, misapplied, or distorted by powerful interests if policy-makers do not meaningfully incorporate ethics into policy-making.” If equity, solidarity, maximizing benefits, global justice, and other ethical values are to serve as key commitments in public health responses, ethics cannot be an afterthought.^13^

Ethics must be a starting point and be meaningfully respected, understood, and incorporated into policy making. There is a need to build competence and capacity of and in the public health workforce through education and training, and for public health leaders to highlight and courageously stand up for public health values. Also, in the same way that epidemiologists and scientists are involved in decision making, it is imperative that ethicists be included in all stages of the decision-making process.

## Building ethico-legal competence, capacity, and resilence

The theme of the 17th World Congress of Public Health, held in Rome in May 2023, was: *A world in turmoil—opportunities to focus on public’s health*. The Congress provided opportunity to hold a number of sessions on public health ethics, values, and law. There was recognition of the central importance of the issue, and the need to build a coalition to further advance the discourse, scholarship, and practice of public health ethics and law.

It was agreed at a workshop sponsored by the Global Network for Academic Public Health, in collaboration with the European Public Health Association, the Association of Schools of Public Health in Europe, the International Association of National Public Health Institutes, and the UK Faculty of Public Health, and other partners that the priorities for the coalition would include: the mission and mandate of public health, creating a code of ethics and professional conduct for public health institutions and professionals, building ethico legal competence and capacity through education and training, moral distress and injury of health workforce, law and ethics for climate action, indigenous knowledge and world views, Health Peace Practitioners. It was also agreed to further strengthen the coalition and partnership through the Public Health Ethics and Law Network and to develop a community of learning and collaboration to take forward the agenda.

## Public health ethics and law for good public health policy and practice

On 18 June 2025, there was a significant international roundtable summit on *Public Health Ethics, Values and Law for Good Public Health Policy and Practice*, held at the Royal College of Physicians in London. Organized by the European Public Health Association, the Global Network for Academic Public Health, the Association of the Schools of Public Health in Europe, and the Faculty of Public Health through the Public Health Ethics and Law network. It brought together ethico-legal scholars, academics, and senior public health practitioners, and leaders from a range of public health organizations from around the world. The purpose of the summit was to reflect, consult, and develop lines of action to take forward thinking, and work on the various issues agreed at the World Congress, and further develop the community of learning and collaboration.

At the Summit in London, there was stark realization of the turbulence of the times. The Head of Public Health from Ukraine, senior public health colleagues from Israel, and Sudan could not attend due to conflict in their countries. A colleague from the US, who was not an American citizen, had feared leaving their university in the US, in case they were perhaps not able to return. A colleague from Africa noted the recent significant abrupt cuts in funding and implications for public health programs and research. There was concern about the growing populism, geopolitical situations, and destructive narratives with significant implications on the health of people, the planet, and our futures. A plaque was placed at the foot of an olive tree in the prestigious historic medicinal plant gardens at the Royal College of Physicians, marking the summit, with lines from the special message to the Summit in London from Dr Tedros Adhanom Ghebreyesus, Director General of the World Health Organization, that “Peace is the best medicine … without peace there can be no health.”

This supplement of the *Journal of Public Health*, on *Ethics, Values, and Law for Public Health in a World in Turmoil*, is a further contribution to advancing the agenda to advance the discourse, scholarship, and practice in public health ethics. It is a collection of papers, commentaries, and perspectives from around the world resulting from a general call for papers on the issue. A number of manuscripts cover the issues raised at the summit, providing knowledge and insights for the public health ethics and law community of learning and collaboration to further advance the agenda.

At this inflection point in history we cannot be passive observers. There is a need for resilience, solidarity and leadership to stand up for our values and commitments for better health for all, social justice, and betterment of the world. The individual, community, and institutions which serve us are key protagonists and chief stewards for health and it is through their interactions and values that the future emerges. There is the need for global solidarity, justice and empathy, and the case to reimagine the earth as one country and humankind its citizens.

“We are not tinkers who merely patch and mend what is broken... we must be watchmen, guardians of the life and the health of our generation, so that stronger and more able generations may come after.” Dr Elizabeth Blackwell (1821–1910), The First Woman Doctor.

Dr Farhang Tahzib, Convenor, Public Health Ethics and Law Network; President, Ethics in Public Health Section, European Public Health Association. farhang.tahzib@gmail.com

## Data Availability

United Nations, September 2021. https://www.un.org/ en/content/common-agenda-report/

## Notes

**Farhang Tahzib**, Public Health Ethics and Law Network, Global Network for Academic Public Health and President, Ethics in Public Health Section, European Public Health Association
